# Selections

**Published:** 1817-04

**Authors:** 


### PART III.

### SELECTIONS.

—— Factorum est copia nobis.

### FOREIGN JOURNALS.

#### *Cases of Fracture of the Patella produced by muscular Action and cured without Bandage.**

First Case.—Louis Levrat, aged 45, on the 27th of September 1810, fell upon his side; so that the right leg, violently bent, suddenly sustained the whole weight of the body. No pain was at

* Observations sur les fractures de la rotule produites par l'action musculaire first felt; and impracticability of rising, alone announced the existence of the injury. He was immediately conveyed to *L'Hotel Dieu.* On the morning visit, a transverse fracture of the patella, about one third from the superior margin, was readily detected. During the first twenty days, the swelling, both œdematous and inflammatory, which affected the joint, was alone combated. The leg was preserved in permanent extension by means of splints so disposed as to form from heel to hip an inclined plane, upon which the whole limb slightly bent on the pelvis rested. This position alone sufficed to maintain in accurate contact the fragments of the patella. Nevertheless, on October the 13th, M. Pelletan, with a view of farther extending the leg, applied a bandage which the patient either could not or would not bear. M.Giraud substituted another for it on the day after, but without better success. On the 16th, he had again recourse to the means which had answered so well when the swelling of the articulation forbade the employment of a bandage; and by these alone the consolidation of the fracture was nearly complete on the 5th of November, and, at the end of the month, there existed only an interval of about three lines between the re-united fragments of the bone. At this period, Levrat was able to leave the hospital on foot.

Second Case.—In the beginning of July 1810, a man, aged 22, fell in running; and both knees, particularly the right, came violently in contact with the pavement. A smart pain with slight swelling came on; and he was obliged to rest for eight days. But these symptoms gradually abated, or were only felt at intervals on undue exertion in walking; when on the 8th of October following, he received the injury which forms the subject of the narrative.

In descending a narrow stair, both heels slipped and he fell some steps lower down with both legs bent under him. At the same instant, he felt something give way in the right knee, which was soon affected with a painless swelling. He bound his handkerchief tightly round the joint, and thus contrived to reach a hackney-coach, which conveyed him directly to *L'Hotel Dieu;* where, according to his own statement, he walked almost without assistance to the appointed ward. On examination, a transverse fracture of the patella was discovered near its middle, complicated with considerable tumefaction of the adjacent parts. Compresses were applied, and the limb, slightly bent upon the pelvis, was kept extended as in the preceding case; so that the moveable fragments of the patella were preserved in close contact. Within eight days, the swelling of the joint had totally disappeared. By the end of the month, there was no longer any motion between the fractured portions. Lastly, the patient, after exercising himself for a time, both with and without crutches, was dismissed on the 1st of December, perfectly cured, with the exception of a considerable stiffness of the joint.

At the time these cases occurred, I saw, in the same ward, a man who, eight years previously, had fractured the patella, as was evident from a transverse groove in the middle of the bone. He assured me that the bone-setter, to whom he applied, did nothing more than keep the leg constantly extended on the thigh by means of a bandage, which never at all prevented him from walking about, even in the streets of Paris.

*Reflections on the preceding cases, extracted from a report made to l'Athénée de Médecine. By Dr. Moreau.* It has been long known that muscular contraction is sufficient to produce the fracture of certain bones, more particularly the patella. This truth, established on numerous and incontrovertible facts, is no longer disputed. Not so with the treatment which such injuries require. Some surgeons, contenting themselves with extending the leg on the thigh and bending the latter on the pelvis, leave to Nature the consolidation of the fracture. Others assist the position by the action of an appropriate bandage. This difference of treatment seems to be highly important, if we may judge from the result. Let us suppose, for a moment, that the fragments of the fractured patella can only be re-united by an intermediate fibrous substance; and we will briefly examine the advantages and incoveniences, respectively attached to the two methods of treatment.

When position is merely employed, as in the cases communicated by Dr. Lens, the patient escapes the pain resulting from the application of a bandage. But what is the consequence? Tumefaction, œdematous or sometimes becoming inflammatory, attacks the soft parts which surround the joint; and commonly continues, in some degree, during the treatment of the fracture. This tumefaction prevents the fragments of the bone from being placed in immediate contact; and, even when it is insufficient to produce this effect, the extensor muscles of the leg, not being counterbalanced in their action, contract and draw up the superior fragment of the bone, so as to produce a separation. If consolidation be, under these circumstances, effected, the fibrous substance, uniting the portions of bone, will be three or four lines in length; and when, at the end of fifty or sixty days, the patient begins to walk, this substance, being thin, will stretch, increase the length of the tendon, and thus singularly diminish the power of the muscles inserted into it. Progression becomes difficult and unsteady, and the patient is liable to frequent falls.

But when to suitable position is added the application of a proper bandage, great advantages result to the patient. In fact, if this bandage be applied at the moment of the accident, it prevents, or at least moderates, the tumefaction of the soft parts. But if some days have elapsed, and inflammatory symptoms already exist, we should wait till they are dissipated; and then the application of the bandage will be little, if at all, painful. The kind of œdema, which succeeds inflammation, is speedily removed by it. The fragments of the patella are preserved in immediate contact. The muscles of the thigh, compressed on all sides, can no longer act to produce separation. Re-union is accomplished. The intermediate fibrous substance, only a line or two in extent, and thick, yields not to the action of the muscles; and the powers of progression and keeping the erect posture are unimpaired. In addition to these advantages, resulting from the use of a bandage, there is another, never to be obtained by simple position; that is, immediate re-union. Although the patella be destitute of periosteum, and Nature cannot form in this as in other bones, a temporary callus whereby to connect the fragments until the permanent callus is firmly established, it is now clearly demonstrated that the fractured patella may be immediately consolidated without the intervention of the fibrous substance. Professor Lallemand, some years since, presented to the Faculty of Medicine of Paris a patella which had been once fractured, and exhibited an example of re-union by a real boney callus. A patella, displaying the same phænomena, is possessed by Professor Dupuytren. But, in order to obtain this direct re-union, it is necessary that the patient should be confined to rest in the apparatus for ninety or a hundred days. I have seen several cases of fractured patella thus treated by Professor Dupuytren, and cured without the intervention of the fibrous substance or anchylosis. There only remained, for some time, stiffness and difficulty of motion of the knee, as invariably happens to individuals who have been cured by simple position.

Yet, must we not suppose, that all fractures of the patella are susceptible of such treatment. It is evident, that if a bandage be applied in cases where the bone is broken into small pieces, where there is effusion and great disorder of the joint, we shall expose the patient to many inconveniences. But I think that all fractures of the patella produced by muscular action, all which are unattended with great contusion or effusion, should never be left to Nature; that in such, both position and bandage should concur in the accomplishment of a cure as perfect as possible.

---

### FRENCH DICTIONARY OF MEDICAL SCIENCES.

( Continued from page 76. )

MEDICAL JURISPRUDENCE. *Article*, *Blessure* (continued).—Wounds may become either a proximate or remote cause of death; and, on this principle, the different degrees of mortality have hitherto been founded. We shall not pause to examine the several divisions of wounds, proposed by various writers, their accuracy or defects. The following is that adopted by M. Marc. He divides lesions into two classes, the *mortal* and *not mortal*. The first of these comprehends two orders, the *necessarily mortal* and *accidentally mortal;* and the last order, two genera, the *directly* and *indirectly mortal*. Under the second class are also included two orders, the *perfectly* and the *imperfectly* curable.*

* Foderé, in his last edition, divides wounds into the *necessarily mortal, accidentally mortal; necessarily dangerous, accidentally dangerous;* and

To this arrangement, it may be objected, that wounds of the second class may be confounded with those belonging to the second order of the first. But such defect is inevitable, and the objection more specious than solid. For no lesion can be pronounced mortal, unless death have ensued; and, in this respect, lesions of the second class can never be confounded, in practice, with those of the first, although several species may belong equally to both divisions.

From the impossibility of considering lesions abstractedly in juridical medicine, it always happens that they who, *à priori*, attempt to distinguish and class the species, are guilty of inaccuracy. We should be content to establish the classes, orders, and, at most, the genera; and leave the juridical practitioner to class individually the species, as they present themselves. Such, at least, is the opinion of Mahon: and Foderé, in attempting to discriminate the species, has committed the error to which we allude. Yet, notwithstanding the impracticability of distributing, *à priori*, the different species under their respective orders, let us attempt to particularise some, with a view to exemplify the application of our principles. Let a complete division of the trachea, with lesion of the principal arteries be supposed; a twisting of the cervical portion of the spine, or the wound of a large and inaccessible artery: these are lesions of the first order of the first class of the division proposed, or wounds necessarily fatal. Contused wounds of the head, fractures of the skull in inaccessible parts, incomplete division of the trachea, slight wounds of the lungs or abdominal viscera, rupture of an aneurism, or internal abscess from external violence, constitute so many species of the first genus under the second order of the first class, or wounds directly accidentally fatal. Superficial wounds, slight contusions, or other injuries, which, from a diseased constitution or other unfavourable circumstance, prove ultimately fatal, are examples of lesions belonging ing to the second genus of the second order. A principal objection which may be started against this classification is, that lesions, frequently, but not invariably fatal, are thus confounded with the individually fatal, under the common title of lesions directly accidentally fatal: yet, from this method, no serious inconvenience results. Doubtless, the man who inflicts a wound on the abdomen of another, which, though not directly, may eventually, become fatal, is more culpable than one who has the misfortune to kill with a blow his antagonist affected with aneurism: and a juridical report, framed on the preceding principles would, it may be urged, present the accused person under an aspect equally unfavourable. But by the juridical physician, the *corpus delicti* ought alone to be considered: the moral circumstances of the case only concern him,

---

*slight.* By Dr. Male, they are ranged in four classes: the *mortal*, *dangerous*, *accidentally mortal*, and *certainly not mortal.* This latter arrangement is, perhaps, the best.—EDIT.

inasmuch as they have direct relation with the physical state of one of the parties interested.

The second class of lesions, or those not mortal, presents not the same difficulty in the establishment of the species; for they belong exclusively to one or other of the two orders composing the class; and, being more constant in their phænomena, are less difficult of appreciation.

The *lesions curable without derangement of function* are those which constitute the second order of the second class of Foderé,* or *wounds without any danger.* He ranges them in three species: those which penetrate only the common integuments, and are not on the face; those which follow the direction of the muscular fibres, and implicate neither nerves, fasciæ, nor tendons; and, lastly, all such as only require for their cure, exclusion from the air, and the aid of the uniting bandage.

As to lesions curable with derangement of function, their species may be decided by the different kind of function injured. Under each of these, we shall place such a number of examples, as will enable any one to class the different cases.

*Lesions curable with derangement of function.* — *Digestion.* Loss of several teeth, particularly incisors; paralysis of one or more masticatory muscles; salivary fistula; enfeebled action of the stomach and intestines; artificial anus; hernia; incontinence of urine. — *Absorption.* Disordered chylification, attributable to the preceding causes. — *Circulation.* Varices; aneurisms. — *Respiration.* Asthma; enfeebled lungs.—*Secretion.* Glandular tumours; cysts; scirrhi.—*Nutrition.* Atrophy of one or more organs.—*Sensation.* Injury or loss of vision; palsy of the eye-lids; deafness; loss of an ear or nose; chronic pains, induced by atmospheric variations; loss of memory; weakness or perversion of intellect. — *Motion.* Diminution, weakness, or destruction, of certain faculties; loss of a limb.— *Speech.* Paralysis of the tongue; aphonia; dumbness.— *Conception and generation.* Prolapsus of the womb; loss of the *membrum virile*, or one or both testes; paralysis of the erector muscles; loss of a mamma, or destruction of its office.

In order to determine the degree of danger or fatality of an individual lesion, it is requisite to consider briefly the three following circumstances:

I. *The species of lesion with respect to the wounding cause.* — In *wounds with solution of continuity*, their size, form, depth, direction, and complication with other lesions, are so many points claiming the attention of the juridical physician. The danger of such wounds is chiefly determined by the number and importance of the vessels implicated, and the degree of consequent hæmorrhage. Incised wounds are, in general, less dangerous than the

---

* Foderé, in his last edition, has very judiciously rejected the "methodic classification of wounds," inserted in the first, as being alike unnatural and destitute of utility. Edit.

punctured; because the latter penetrate more deeply, and oppose, by their straitness, the escape of the product of suppuration; and, moreover, only lacerating or imperfectly dividing the tendinous and nervous parts, they often excite more violent sympathetic disorder. Contused wounds, and especially the gun-shot, are the most dangerous; not only because they deaden and destroy the injured part, and cause commotion of those in the vicinity, or of the whole system, but because they frequently induce consecutive hæmorrhage, and, in proportion to the foreign bodies which they contain, readily excite inflammation and suppuration, so severe as to exhaust the patient's strength. Again, they are more liable than others to the attack of sphacelus. The danger of poisoned wounds can only be appreciated, from a knowledge of the substance introduced.

*Contusions* are complicated, or not, with solution of continuity. The parts injured may, according to the violence of the blow, be weakened, or deprived of life. The blood stagnates in the vessels, is effused under the skin, and thus forms bruises, which, however, must not be confounded with extravasations independent of an external cause. Bruises are especially dangerous, as being frequently accompanied by commotion of important organs, and succeeded by violent inflammation, suppuration, or even sphacelus.

The danger of *commotions* varies according to the importance and susceptibility of the organ affected. Violent concussions of the brain are those most directly fatal. The internal traces of these lesions frequently elude anatomical research; but, sometimes, ruptures, hæmorrhage, inflammation, suppuration, or sphacelus, is discovered. Commotions of the muscles, nerves, and vessels, weaken and derange their functions.

The danger of *burns* is proportionate to the action of the free caloric; which may have merely produced great irritation of the parts attacked, or destroyed their organic structure. It depends, also, on their extent, depth, and especially on the nervous susceptibility of such parts.

*Freezing* causes suspension of life in the parts affected; and the danger principally results from the mode in which the first remedies are administered: inflammation and sphacelus are the consequences of a too sudden transition from cold to heat.

*Dislocations* and *fractures* present more or less danger, according to the situation of the injured part; to their simplicity or complication, and to the severity of the attendant symptoms.

When lesions are not immediately fatal, the state of inflammation is the principal point to be attended to in judging of their danger. This is to be appreciated by the degree and extent of such inflammation, the importance of the inflamed organ, and the possibility of preventing or removing the morbid action. Sometimes it is essential to determine whether gangrene might have been obviated; whether the suppuration be proportioned to the strength of the patient; and whether it have been possible to procure an issue for the pus.

(To be continued.)

